# An objective, markerless videosystem for staging facial palsy

**DOI:** 10.1007/s00405-021-06682-z

**Published:** 2021-03-15

**Authors:** S. Monini, S. Ripoli, C. Filippi, I. Fatuzzo, G. Salerno, E. Covelli, F. Bini, F. Marinozzi, S. Marchelletta, G. Manni, M. Barbara

**Affiliations:** 1 ENT Clinic, NESMOS Department, Faculty of Medicine and Psychology, Sapienza University, Rome, Italy; 2 Laboratory Unit, Sant’Andrea University Hospital, Via di Grottarossa 1035, 00189 Rome, Italy; 3 Department of Mechanical and Aerospace Engineering, Faculty of Civil and Industrial Engineering, Sapienza University Rome, Rome, Italy

**Keywords:** Facial palsy, Subjective grading system, Objective grading system, Videorecording, Smartphone, Machine learning

## Abstract

**Purpose:**

To propose a new objective, video recording method for the classification of unilateral peripheral facial palsy (UPFP) that relies on mathematical algorithms allowing the software to recognize numerical points on the two sides of the face surface that would be indicative of facial nerve impairment without positioning of markers on the face.

**Methods:**

Patients with UPFP of different House–Brackmann (HB) degrees ranging from II to V were evaluated after video recording during two selected facial movements (forehead frowning and smiling) using a software trained to recognize the face points as numbers. Numerical parameters in millimeters were obtained as indicative values of the shifting of the face points, of the shift differences of the two face sides and the shifting ratio between the healthy (denominator) and the affected side (numerator), i.e., the asymmetry index for the two movements.

**Results:**

For each HB grade, specific asymmetry index ranges were identified with a positive correlation for shift differences and negative correlation for asymmetry indexes.

**Conclusions:**

The use of the present objective system enabled the identification of numerical ranges of asymmetry between the healthy and the affected side that were consistent with the outcome from the subjective methods currently in use.

## Introduction

Objective systems for grading unilateral peripheral facial palsy (UPFP) started to be proposed with the aim to overcome the several flaws revealed by the traditional, subjective methods. If it is true that these latter methods, such as the House–Brackmann or HB [1] or the Sunnybrook or SBGS [2] method, fail to highlight some important features of a facial impairment or its sequel, it is also true that the use of software-based systems could at times be difficult to use and, although objective and accurate [3–9], could also fail to consider all the aspects of the facial disfiguration, both with two- and three-dimensional methodologies [10–14]. In this regard, systems sensitive to global or partial changes of the face or to the presence of synkineses or secondary defects changes have still to be introduced in clinical practice.

In an attempt to elaborate an objective method for this purpose, an automatic software-based system has previously been reported and validated for the evaluation of UPFP via the analysis of shifting of markers preliminarily placed on specific face regions [15, 16]. Although the system was appropriate for most UPFP cases and also consistent with the outcome from the subjective HB grading system, it is likely that marker placement may represent a biasing variable that is conditioned by the examiner’s experience and by the different physiognomic characteristics of some affected individuals presenting with a poorly defined eyelid region, especially during closure and opening of the eyes.

The purpose of the present study was to elaborate a video recording automatic system for grading patients with UPFP without using facial markers. In this regard, the study group was composed of subjects who were previously classified and validated with another objective video recording system [15, 16].

## Materials and methods

Forty subjects affected by UPFP were recruited for the present study. The subjects were consecutively included in each corresponding HB stage, from II to V, with ten subjects per group. All these subjects were previously assessed by a marker-based system [15] that provided data significantly correspondent to those obtained via both HB and Sunnybrook grading systems [16].

The main steps of the marker-based procedure were as follows:

- Placement of markers in both sides of the face at the levels of the upper, medium and lower sectors;
- Capture the frontal view of the subjects’ face via video using a smartphone camera with flash on to enhance marker reflectivity.

Video recording lasted 15/20 s for each patient. The patient was first asked to remain still and then to perform five common facial expression, including frown the forehead, mild eye closure, strong eye closure, smile and kiss, returning to the resting position after each movement.

**Fig. 1:**
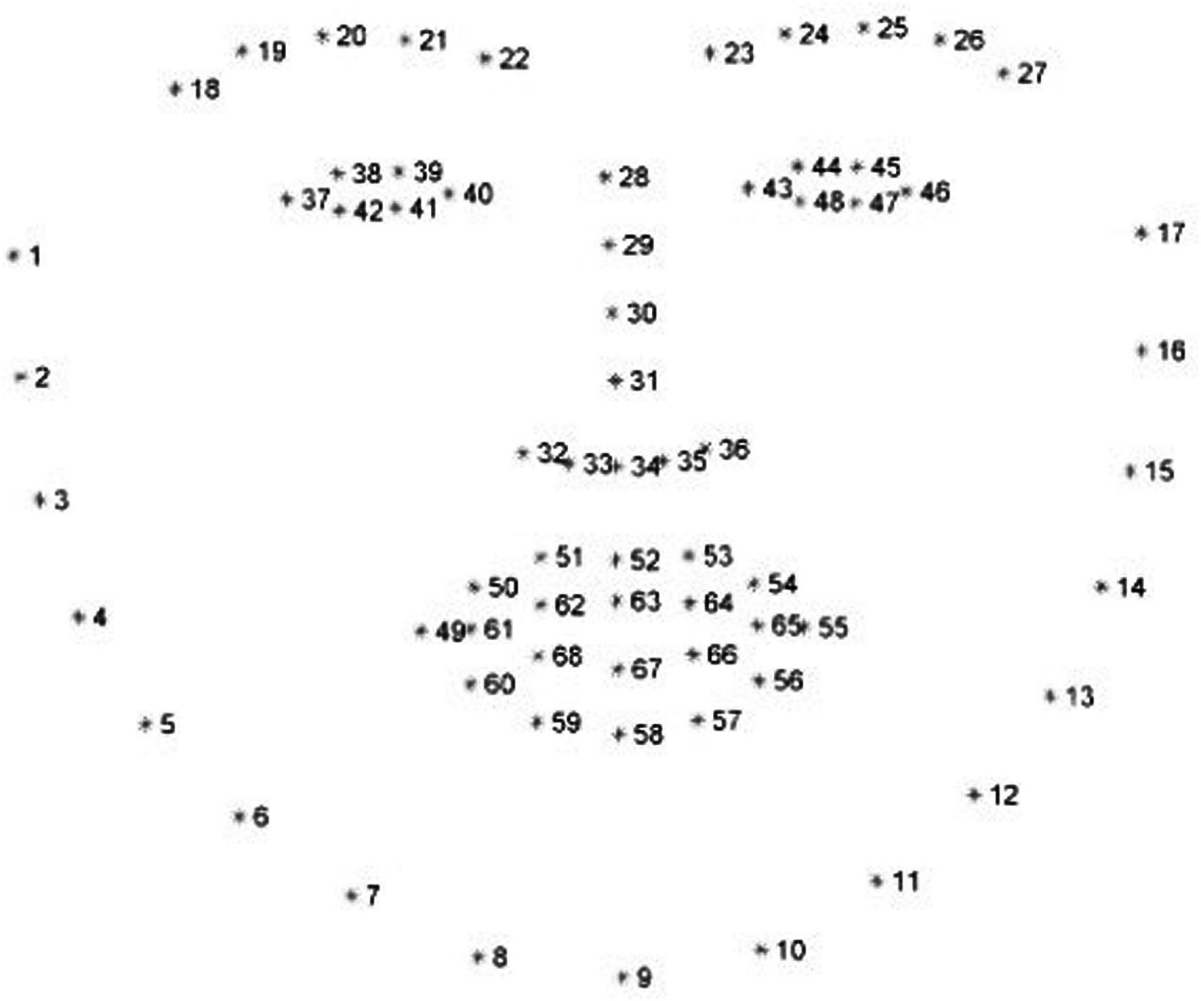
Numbers related to specific points of anatomical facial structures individuated by a machine learning algorithm [18]

In the present study, a new automatic system was applied to the same group of patients, enabling the tracing of face points without positioning of the markers. This procedure starts with the individuation of the facial points using a machine-learning algorithm that allows the automatic individuation of the face points [17]. By doing so, a ROI (region of interest) is added to the algorithm. This algorithm is based on a Histogram of Oriented Gradients (HOG) combined with a vector machine of support to obtain a plane containing the patient’s face. Subsequently, using an algorithm based on “1 ms face alignment” with an ensemble of regression trees [18], 68 facial points were identified (Fig. 1). The identification of these 68 points was derived by decisional trees trained on one thousand face images in movement manually individuated. Therefore, the algorithm individuated the 68 points on the ROI, yielding the coordinates in pixels with a 68-line matrix (one for each point) and 2 columns (one for the *x*-axis, the other one for the *y*-axis). Before the elaboration, the measurements were converted from pixels into millimeters, considering 5 mm for each marker given that the markers were obtained by an automatic sheet-punching machine, and the dimensions of the markers in pixels on the image, based on the following formula:

$$ t = \frac{f[mm]}{{f[px]}}. $$

**Fig. 2:**
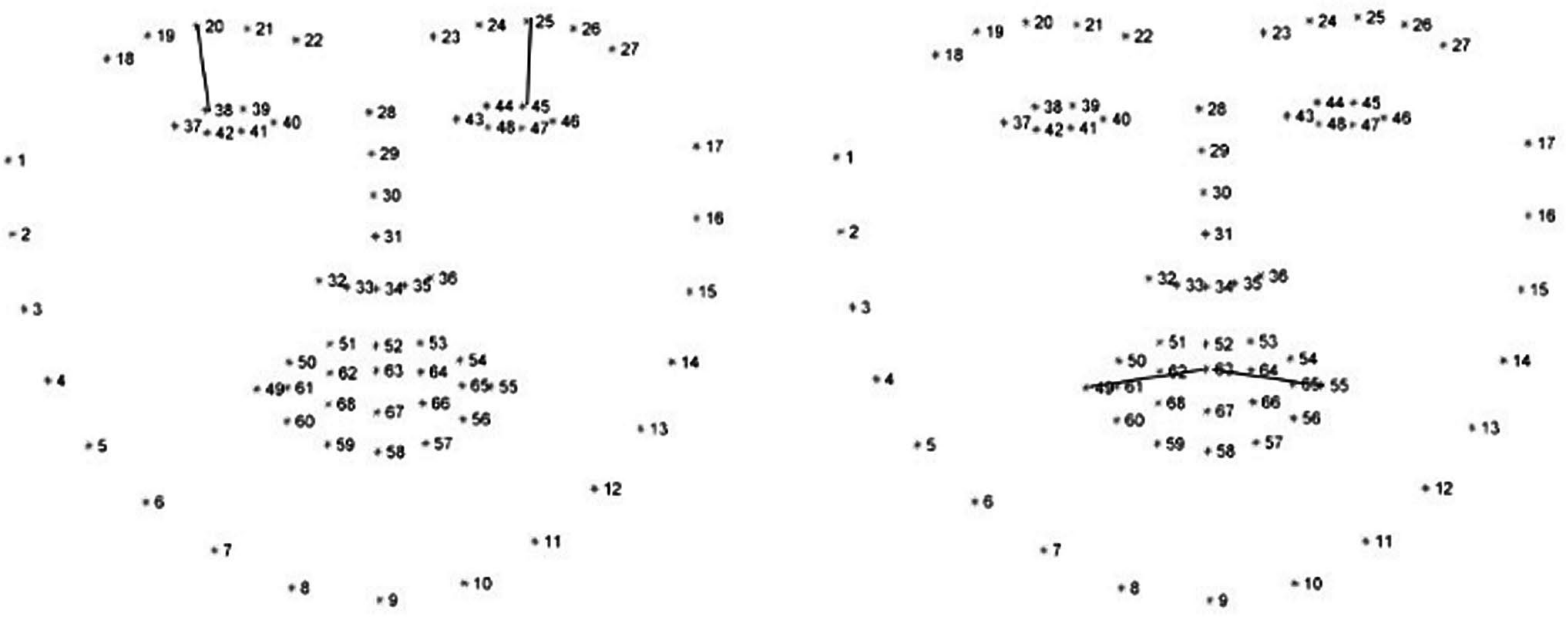
Points to identify on the face the distances (continuous line) between eyebrows-upper eyelids (left) and the mouth corners (right) in the healthy and the affected side, individuated by “decisional trees” [19]

The following points were considered significant (Fig. 2):

- 20 and 25 for the eyebrow region
- 38 and 45 for the upper eyelid rim
- 42 and 47 for the lower eyelid rim
- 49 and 55 for the mouth corner
- 63 center of the mouth
  $$ \left( {\begin{array}{*{20}c} {x_{1} } & {y_{1} } \\ \cdot & \cdot \\ {x_{68} } & {y_{68} } \\ \end{array} } \right). $$

Afterward, the video elaboration began with the window “import video” that uploaded all the videos, selecting the frames of interest. Once obtained, these data were reported in graphs using the module “matplotlib” and memorized on a.csv (comma-separated values) file accessible by Excel.

By doing so, the software delivered graphs and numerical data for each ROI on a specific frame window. It was, therefore, possible to perform a quantitative assessment of UPFP by evaluating the eventual shift of the selected point for the healthy side in comparison of that of the affected side, in patients who were already classified and validated in a previous study [16].

Two face movements (forehead frowning and smiling) were analysed in the normal and in the affected side, comparing the marker and the markerless methods.

Initially, the maximum distances of the eyebrow shift from the eyelid in the forehead frowning and the shift of the mouth corner from the mouth center during smiling were calculated in both sides of the face. To provide reliable quantitative values of the real shift for each side, the mean distance at rest was subtracted from the maximum distance during each movement.

The second step involved calculating the shift differences between the healthy and the affected side for each movement in patients with different HB grades. As result, mean partial and total values of specific shift differences for each HB grade were obtained.

The last step involved the assessment of the shifting ratio between the affected and the healthy side, using the former as a nominator and the latter as a denominator, to derive the asymmetry index between the two face sides. Similarly, for the asymmetry index both for partial and total scores of the two face movements considered, specific ranges for each HB group of patients were drawn.

### Statistical analysis

Continuous data were summarized by mean and standard deviations (SD). The comparison of the shift difference and asymmetry index between HBII, HBIII, HBIV and HBV was evaluated using a one-way ANOVA test. The *p* values are two sided, and a *p* value ≤ 0.05 was considered statistically significant. All computations were performed using R version 3.5.3 (2019–03-11)—“Great Truth” Copyright © 2019 The R Foundation for statistical Computing and Graph Pad Prism vers. 6.01.

## Results

**Fig. 3:**
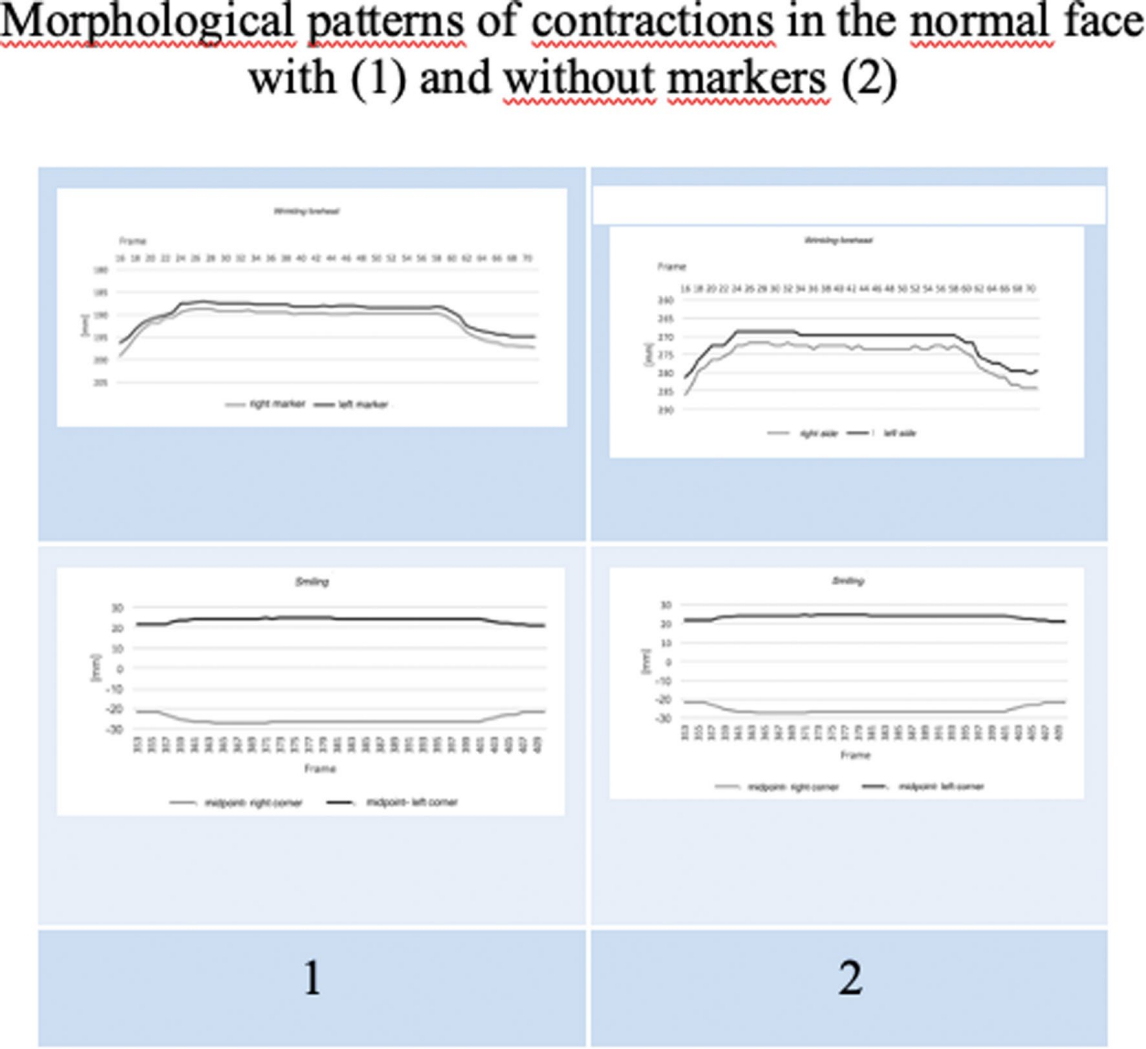
Morphological comparison between marker [1] and markerless [2] point shifting in the normal face during forehead frowning and smiling

**Fig. 4:**
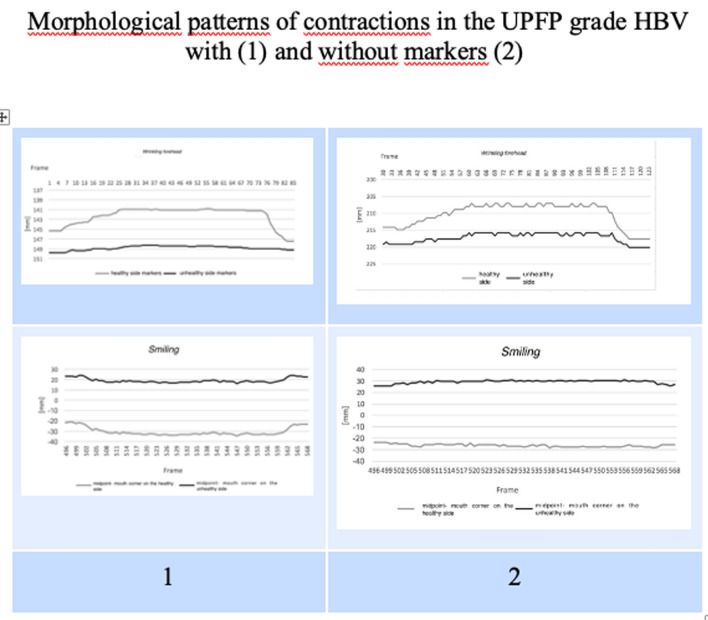
Morphological comparison between marker [1] and markerless [2] point shifting in the paralyzed face of HBV grade patients, during forehead frowning and smiling

The morphological curves of the two movements (forehead frowning and smiling) in both the normal and the UPFP situation derived from the two methods of analysis with and without reflective markers were comparable (Figs. 3, 4). The partial values of the shift differences of eyebrow elevation in the vertical plan when frowning the forehead ((shifts of the eyebrow from the eyelid) and in the horizontal plan when smiling (shift of the two mouth corners from the center of the upper lip) between the healthy and affected side for each subject belonging to HB grade II–V are shown in Table 1 a and b, respectively. The partial range specific for each HB grade of the shift differences was as follows:

- Eyebrow elevation was 0.40–2.86 (mean 1.65) in HBII; 3.76–4.46 (mean 4.03) in HBIII; 4.54–5.81 (mean 5.20) in HBIV; and 6.33–11.07 (mean 8.18) in HBV.
- Smiling was 0.32–2.75 (mean 1.34) in HBII; 3.00–3.90 (mean 3.37) in HBIII; 3.99- 4.65 (mean 4.25) in HBIV; and 4.99–6.24 (mean 5.56) in HBV.

**Table 1:** a, b: Partial shift differences between healthy versus affected side in each HB grade during vertical (forehead frowning) and horizontal (smiling) face movements: the mean shift differences increase significantly with the HB grade increase (*p* ≤ 0.0001)

Shift differences in the healthy and affected side during forehead frowning					
Score	HB II	HB III	HB IV	HB V	
Patient 1	0.87	3.89	5.56	6.93	
Patient 2	0.61	4.24	5	11.07	
Patient 3	2.81	4	5.23	6.5	
Patient 4	1.66	3.8	4.54	6.32	
Patient 5	0.39	3.75	5.26	6.65	
Patient 6	2.53	3.95	4.82	7.60	
Patient 7	1.58	3.87	5.01	10.23	
Patient 8	2.86	4.23	4.96	8.65	
Patient 9	1.89	4.12	5.78	9.48	
Patient 10	1.26	4.45	5.80	8.32	
Mean	1.65	4.03	5.20	8.18	
Max	2.86	4.46	5.81	11.07	
Min	0.40	3.76	4.54	6.33	
St. dev.	0.89	0.23	0.42	1.67	

Shift differences in the healthy and affected side during smiling					
Score	HB II	HB III	HB IV	HB V	
Patient 1	0.93	3.00	4.56	6.00	
Patient 2	2.72	3.13	3.99	5.33	
Patient 3	2.33	3.65	4.24	5.00	
Patient 4	1.65	3.11	4.65	5.24	
Patient 5	0.64	3.31	3.99	6.13	
Patient 6	0.98	3.13	4.32	5.13	
Patient 7	1.33	3.57	4.04	4.99	
Patient 8	0.32	3.90	4.02	5.55	
Patient 9	0.82	3.69	4.56	5.99	
Patient 10	1.73	3.26	4.11	6.24	
Mean	1.34	3.37	4.25	5.56	
Max	2.72	3.90	4.65	6.24	
Min	0.32	3.00	3.99	4.99	
St. Dev.	0.76	0.30	0.26	0.48	

The statistical comparison of the shift difference ranges in the different HB grades exhibited a significant difference among each HB grade (*p* ≤ 0.0001) both for the partial and total values. ANOVA tests showed that the mean difference during forehead frowning increased significantly as the HB grade increased (*F* = 78.02; *R*^2^ = 0.90 (Fig. 6a) and that the mean shift difference during smiling increased significantly during smiling as the HB grade increased (*F* = 107.7; *R*^2^ = 0.92) (Fig. 6b).

The total scores of shift differences between the two face sides are summarized in Table 2 and have been 1.03–5.13 for HBII; 6.89–8.13 for HBIII; 8.98–10.35 for HBIV; and 11.49–16.39 for HBV.

**Table 2:** Absolute and mean values of the shift differences of the total facial movements in each HB grade

Total shift differences in the healthy and affected sides				
Score	HB II	HB III	HB IV	HB V
Patient 1	1.80	6.89	10.12	12.92
Patient 2	3.3	7.36	8.98	16.39
Patient 3	5.13	7.65	9.47	11.49
Patient 4	3.31	6.91	9.19	11.56
Patient 5	1.03	7.06	9.25	12.78
Patient 6	3.51	7.08	9.14	12.73
Patient 7	2.91	7.44	9.05	15.22
Patient 8	3.18	8.13	8.98	14.20
Patient 9	2.71	7.81	10.35	15.47
Patient 10	2.98	7.71	9.91	14.56
Mean	2.99	7.40	9.44	13.73
Max	5.13	8.13	10.35	16.39
Min	1.03	6.89	8.98	11.49
St. dev.	1.07	0.41	0.50	1.68

The *p* value for the total scores of the shift differences in the HB grades was significant (< 0.0001). For the total scores of the shift differences, the ANOVA test also showed a strict positive correlation with the HB grade increase (ANOVA test: *F* = 183.0; *R*^2^ = 0.95).

**Fig. 5:**
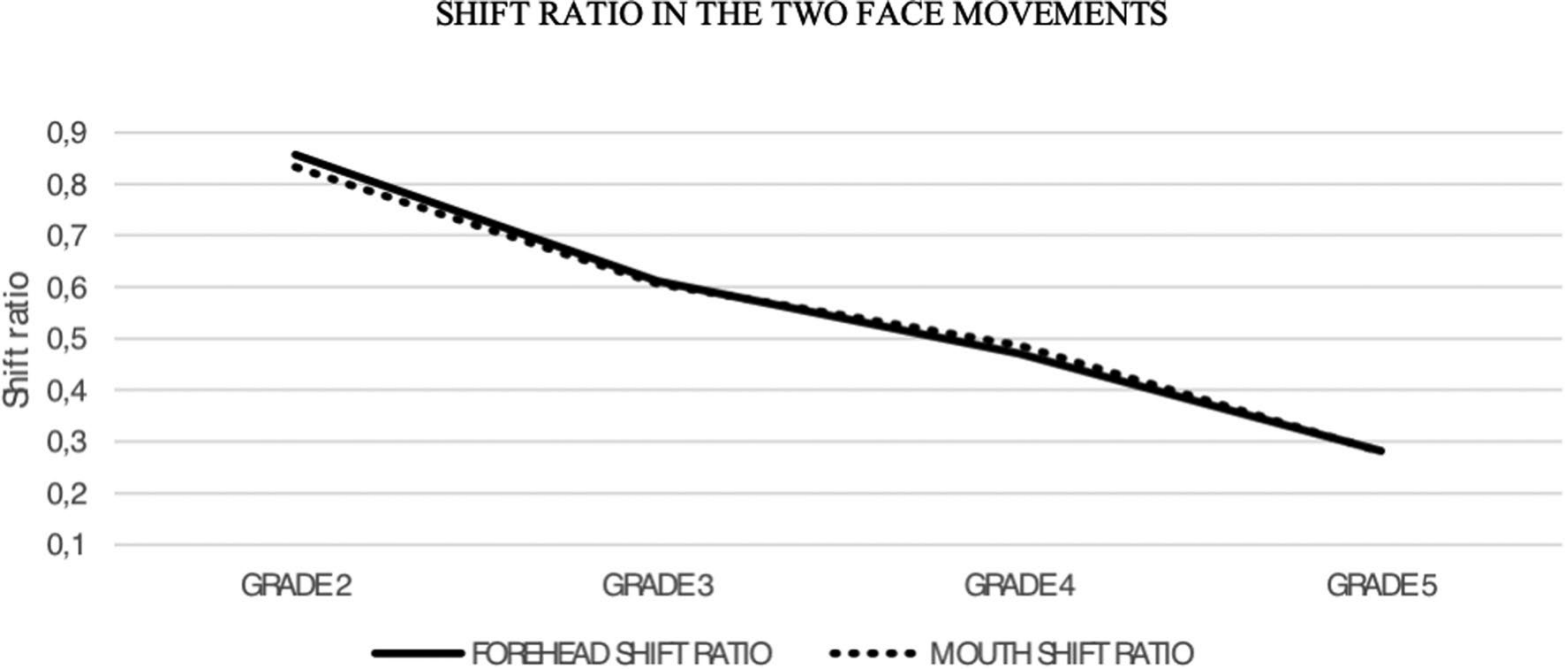
The asymmetry index of the frontal and mouth regions decreases with the HB grade increase

Regarding the shift ratio, the asymmetry indices for the frontal region were in the range of 0.74–0.98 (mean 0.85) in HBII; 0.54–0.68 (mean 0.61) in HBIII; 0.1–0.2 (mean 0.46) in HBIV; and 0.03–0.38 (mean 0.22) in HBV (Table 3a). In the mouth region, the ranges of shift ratio were 0.73–0.94 (mean 0.82) in HBII; 0.55–0.69 (mean 0.62) in HBIII; 0.43–0.51 (mean 0.48) in HBIV; and 0.1–0.34 (mean 0.24) in HBV (Table 3b). The ranges were statistically different for both the partial and the total values (*p* = 0.0001). The ANOVA test showed that the asymmetry index of the frontal (*F* = 96.68; *R*^2^ = 0.91) and the mouth regions (*F* = 220; *R*^2^ = 0.96) decreased significantly as the HB grade increased (Fig. 5). A negative correlation between the mean total scores of the asymmetry index and the HB grade increase was observed (*F* = 207.50; *R*^2^ = 0.96).

**Table 3:** a–b: Partial shift ratio of forehead (a) and mouth (b) regions of all patients in each HB grade: the shift ratio decreases with the HB increase

Shift ratio (asymmetry index) in the frontal region					
Score	HB II	HB III	HB IV	HB V	
Patient 1	0.80	0.55	0.52	0.03	
Patient 2	0.85	0.68	0.50	0.26	
Patient 3	0.98	0.63	0.50	0.16	
Patient 4	0.98	0.65	0.43	0.12	
Patient 5	0.78	0.59	0.47	0.29	
Patient 6	0.92	0.68	0.41	0.18	
Patient 7	0.78	0.54	0.47	0.38	
Patient 8	0.74	0.58	0.44	0.33	
Patient 9	0.76	0.58	0.48	0.32	
Patient 10	0.93	0.64	0.45	0.17	
Mean	0.85	0.61	0.46	0.22	
Max	0.98	0.68	0.52	0.38	
Min	0.74	0.54	0.41	0.03	
St. dev.	0.09	0.05	0.03	0.10	

Shift ratio (asymmetry index) in the mouth region					
Score	HB II	HB III	HB IV	HB V	
Patient 1	0.84	0.64	0.50	0.14	
Patient 2	0.80	0.61	0.48	0.22	
Patient 3	0.77	0.60	0.48	0.25	
Patient 4	0.76	0.61	0.45	0.14	
Patient 5	0.73	0.65	0.50	0.28	
Patient 6	0.94	0.65	0.51	0.28	
Patient 7	0.91	0.69	0.51	0.34	
Patient 8	0.78	0.55	0.50	0.33	
Patient 9	0.84	0.55	0.43	0.10	
Patient 10	0.91	0.68	0.45	0.33	
Mean	0.82	0.62	0.48	0.24	
Max	0.94	0.69	0.51	0.34	
Min	0.73	0.55	0.43	0.10	
St. dev.	0.07	0.04	0.02	0.08

## Discussion

The wide variety of available clinical and objective methods for the diagnosis of UPFP suggests their importance for providing reliable grading classifications for prognostic and therapeutic purposes. The traditional clinical classifications [1, 2] are mostly subjective and related to individual experience. Moreover, they present limitations for defining the mimic deviations of the face in quantitative terms. In particular, in the HBGS classification, the presence of synkineses starts to be included from Grade III on [1], whereas the SBGS assigns a specific partial score to synkineses that is masked by the final sum that includes the partial scores of the static and dynamic situations of the face [2]. The digital systems, mostly based on sophisticated software, do not provide a description of the facial alterations in all their aspects, both quantitatively and qualitatively, apart from being at times difficult to perform and time-consuming.

To date, an objective method that could assess all the qualitative modifications of the face mimic as hypercontraction or synkineses on both the affected and healthy side has not yet been proposed. Although none of the subjects included in the present study showed synkinesis in either side of the face, one may assume it useful to adopt an objective method to confirm the clinical classifications and monitor the sequels from a UPFP via clinical observations.

The objective methodologies proposed for the analysis of the face movement have been based on three main elements: video recording, systems for capturing the face structures under static and dynamic positions, and comparison of the affected and healthy side. Several studies have based their analysis on video recordings of the main face movements traced by reflector markers placed on several points of the face sectors [4, 5, 15, 19]. Recently, one methodology has been validated by demonstrating the significant correlation between both HB grades and HB grades derived from SBGS classification, as well as HB grades derived from the marker analysis [15, 16]. It is important to stress that this procedure may be rapid and easy, but also encompasses some difficulties when placing the markers due to individual physiognomic characteristics of the patient’s face, such as an undefined eyebrow or the presence of a mustache.

A few markerless computing analyses studies have recently been reported [20–23], and three-dimensional techniques have been used to document the facial motions [10–14].

The present study has attempted to develop a markerless automatic system for the analysis of the face movements through the recognition of specific points for each structure of interest. The software was first trained on countless images of face movements of healthy subjects, using the “machine learning” method [17], with the recognition of 68 points of interest manually created via an “ensemble of tree decision” [18].

The strength of the present study is that the markerless analysis has been assessed on the video recordings of the subjects already classified and validated as HB grade in previous studies [15, 16], combining the objective marker analysis with two traditional clinical classification (HBGS and SBGS).

A similar markerless study based on the software learning and tree decision has recently been reported [22]; however, photograms instead of videoclips were used, increasing the risk of not exactly quantifying the point distances during the face movements.

Among the possible merits of the present study it is worth stressing that, in addition to the differences of the distances between the two sides of the face, an asymmetry index between the two face sides for each movement, with scores specific for each HB grade, has been calculated. As a matter of fact, the shift differences between the two sides of the face exhibited range values that were significantly different and not overlapping among the HB grades in both movements. In addition, the partial and total ranges of the shift differences increased with a positive correlation with respect to the HB increase. Moreover, the range values for the asymmetry indices were significantly different and did not overlap among the HB grades, and the partial and total scores of the two movements were negatively correlated to the HB increase, as also indicated by the values from the normal subjects used for validating the previous study, having minimum and maximum values (0.99 and 1.2) greater than the other HB grades [16].

Based on these results, one may assume that the ranges of shift differences and asymmetry indices between the two face sides are sensible and specific for each HB grade considered.

A possible limitation of the present study is the fact that facial function was not evaluated via all possible face movements given that the assessment only evaluated two movements: the movement produced by forehead frowning, such as that noted in an expression of astonishment, and the movement produced when smiling. In particular, regarding the movement related to eye closure, it is known to be clinically important to separate minor (grade III and better) from severe cases (grade IV and worse). These movements were not analysed because it was not possible to define a sensible asymmetry index describing the palsy grade. In fact, it is likely to assume that it is not easy to modulate the entity of the contraction during eye closure, especially in subjects affected by a UPFP, as shown by variable asymmetry indices found in subjects with the same HB grade. This information gap, hence, needs to be bridged by combining the present objective procedure with direct evaluation of eye closure.

The methodology adopted in the present study seems to be valid due to the following reasons. The morphology of the markerless points considered to define the two movements in the affected and healthy sides corresponds to the marker method. Second, the methodology has been applied to video-recordings of subjects classified according to HBGS, HBGS derived from SBGS and HBGS derived from marker analysis [16]. Third, this methodology allowed us to identify, for each HB grade, specific ranges of shift differences between the two face sides and specific ranges of asymmetry index for each HB grade. These ranges were assessed for each movement, both individually and together.

In conclusion, the markerless objective method used in the present study may be useful to implement the conventional clinical classifications, given that it maintains the video information during the analysis time and can provide important data on those movements that the traditional subjective methods are unable to assess.
